# Breeding and Molecular Characterization of Insect-Resistant Transgenic Cotton

**DOI:** 10.3390/plants15101551

**Published:** 2026-05-19

**Authors:** Xiaochun Zhang, Jiangtao Yang, Yuxiao Chen, Mengyu Wang, Xuanming Zhang, Mingni Shen, Shuo Zhang, Zhixing Wang, Xujing Wang

**Affiliations:** Biotechnology Research Institute, Chinese Academy of Agricultural Sciences, Beijing 100081, China; 82101221024@caas.cn (X.Z.);

**Keywords:** transgenic cotton, insect-resistant, molecular characteristics

## Abstract

Cotton is one of the world’s important cash crops and occupies a significant position in agricultural production and the national economy. However, insect pests severely affect the growth, yield and quality of cotton. To ensure high and stable cotton yields, the cultivation of insect-resistant transgenic cotton via transgenic technology can not only effectively reduce the impact of chemical pesticides on crops but also exert excellent control effects against pests such as cotton bollworms. In this study, the plant expression vector pC2300-VEC harboring the target genes *epsps*, *cry1Ac* and *vip3A* was introduced into the genome of the recipient cotton cultivar CCRI 24 via Agrobacterium-mediated transformation. The obtained transgenic cotton plants were subjected to the identification of target genes and target traits, and the insect-resistant transgenic cotton line BrsC35 was ultimately obtained. PacBio sequencing combined with conventional molecular characterization methods was used to analyze its insertion site, copy number and other characteristics, providing a new germplasm for insect-resistant transgenic cotton.

## 1. Introduction

Cotton (*Gossypium hirsutum*), as a natural fiber crop, is not only the primary raw material for the textile industry but also occupies a pivotal position in the global economy and serves as an indispensable strategic material [1,2,3]. However, cotton is frequently infested by various pests such as *Helicoverpa armigera*, *Spodoptera frugiperda* and *Ostrinia nubilalis* throughout its whole growth period from sowing to harvest, which seriously restricts cotton yield and quality [4,5]. Among them, the cotton bollworm is one of the major pests in cotton production, causing an average cotton yield reduction of more than 40% in the 1990s [6]. In recent years, the invasive fall armyworm has established itself in China, posing a serious threat to the normal growth of cotton. With the increasing prominence of cotton pest problems, conventional breeding methods can hardly address this challenge. Therefore, cultivating insect-resistant cotton via transgenic technology has become an effective approach for cotton pest control [7,8,9,10,11].

Since the first *Bt* insecticidal crystal protein gene was cloned in 1981, nearly 200 distinct insecticidal crystal protein genes have been cloned and sequenced to date, which have been widely applied in the research of crop insect resistance improvement. Meanwhile, novel *Bt* insecticidal protein genes are continuously isolated and identified. With the advances in functional genomics and high-throughput gene mining technologies, a large number of functional genes have been identified from extremophilic microorganisms, wild relative species and model plants. These genes confer agronomically important traits such as insect resistance, herbicide tolerance, drought resistance, quality improvement, and salt–alkali tolerance. The most widely utilized genes in insect-resistant transgenic crops are derived from *Bacillus thuringiensis* (*Bt*). Among them, the cry-type insecticidal crystal protein genes and vegetative insecticidal protein (*vip*) genes have gained the most extensive application [12,13].

Transgenic technology is one of the modern biotechnologies with the fastest development speed, the widest application scope and the greatest industrial impact worldwide [14,15,16,17,18]. Through genetic engineering, this technology introduces elite exogenous genes with definite structures and functions, such as insect-resistant and herbicide-resistant genes, into the genome of recipient organisms. It can precisely and targetedly improve crop agronomic traits, enable favorable characteristics to be stably inherited by progenies, and further facilitate the breeding of new crop varieties with superior agronomic performance [19,20]. At present, there are three main methods for cotton genetic transformation, namely the pollen tube pathway method [21,22], the gene gun method [23,24], and the Agrobacterium-mediated method [25,26,27], among which the Agrobacterium-mediated method is the most commonly used method in cotton genetic transformation. In 1987, Umbeck et al. successfully introduced the *nptII* gene and *CAT* gene into Coker 312 and C310 via the Agrobacterium-mediated method, laying the foundation for subsequent research on transgenic cotton [28]. In 1990, Perlak et al. obtained transgenic cotton with significant resistance to lepidopteran pests such as cotton bollworm by modifying the plant-preferred codons of the *Bacillus thuringiensis* (*Bt*) insecticidal protein gene, which laid the foundation for the cultivation of transgenic insect-resistant cotton [29]. According to statistics, cotton insect pests can cause a 15–50% reduction in cotton yield; therefore, solving the problem of cotton insect pests is imminent. Since the commercial cultivation of transgenic cotton in 1996, it has effectively controlled the damage of insect pests to cotton [30,31,32,33].

The cultivation of insect-resistant transgenic cotton presents remarkable social, economic and ecological benefits [34,35]. While ensuring stable yield and income increase of cotton, it can not only greatly reduce the application frequency and dosage of chemical pesticides and lower the risk of pesticide residues, but also effectively avoid potential pesticide poisoning hazards to humans, livestock and poultry during agricultural operations, and alleviate secondary fungal infections caused by pest infestation [36,37,38].

While transgenic crops bring enormous social and economic benefits, their safety has attracted widespread public concern. To ensure the sound industrial development of transgenic crops, countries around the world have established independent safety assessment and management systems in accordance with their national conditions. Accurate molecular characterization of transgenic crops is the core of safety evaluation, which mainly includes the integration pattern of exogenous inserted sequences in the genome of transgenic plants and the genetic stability of exogenous genes. The molecular characteristics of transgenic crops play an indispensable role in the approval, supervision, traceability and labeling management of transgenic cotton. Meanwhile, they also facilitate the screening and breeding of transformant progenies by breeders, as well as their commercial application.

Whole-genome sequencing technology serves as an effective approach to identify gene fusions, sequence rearrangements, DNA insertions and structural variations, which have been validated in a variety of animal and plant species [39,40]. In 2012, Zhang et al. [41] used transgenic cattle carrying a 150 kb human lactoferrin gene as experimental material and obtained the flanking sequences of exogenous genes via whole-genome sequencing. Their study verified that whole-genome sequencing is an efficient method for cloning flanking sequences and identifying insertion sites of exogenous genes in genetically modified organisms [41]. In the same year, Kovalic et al. adopted transgenic soybean as research material and confirmed that genome sequencing technology can be applied to analyze the copy number of exogenous genes in transgenic plants, representing a rapid and efficient strategy for molecular characteristic analysis of transgenic organisms [42]. As an emerging tool for deciphering molecular characteristics of transgenic crops, Whole-genome sequencing technology has been widely applied in molecular characterization of transgenic maize, cotton, soybean, rice, sorghum and other crops [43,44,45].

## 2. Results

### 2.1. Genetic Transformation of Cotton and Screening of Transgenic Plants

**Figure 1:**
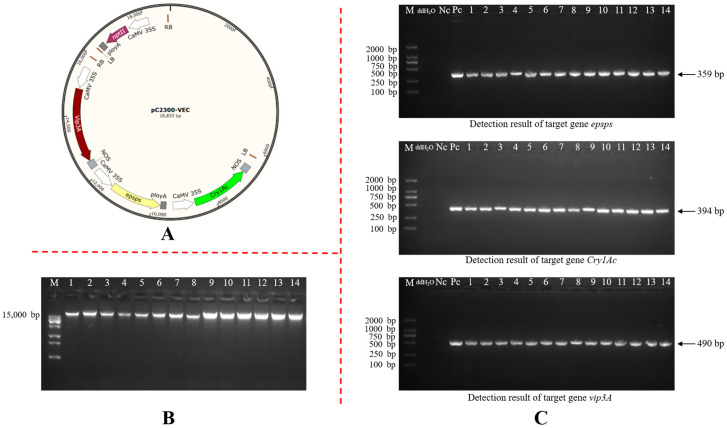
Vector map construction and detection of target genes: (**A**) pC2300-VEC plant expression vector; (**B**) the electrophoresis figure of the cotton genomic DNA; (**C**) PCR identification of the target gene in transgenic cotton; M: DNA marker; 1–14: transgenic cotton plants.

In this study, hypocotyls of aseptic seedlings of the recipient cotton cultivar CCRI 24 were infected with *Agrobacterium* harboring the plant expression vector pC2300-VEC (Figure 1A). After selection with kanamycin, the plantlets were regenerated through stages including callus induction, callus proliferation, and embryogenic callus induction and differentiation. A total of 60 T0 transgenic cotton plants were obtained and designated BrsC01 to BrsC60. Genomic DNA was extracted from leaves of the transgenic cotton plants (Figure 1B), and the target genes were detected by PCR. Among them, 36 plants yielded the expected target bands, indicating that the exogenous genes had been successfully integrated into the cotton genome.

Finally, 36 transgenic positive plants were obtained. Partial detection results are shown in Figure 1C.

### 2.2. Selection of Insect-Resistant Transgenic Cotton Plants

**Figure 2:**
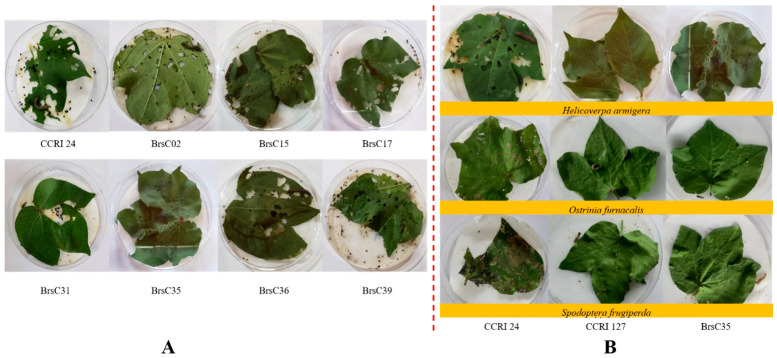
Insect resistance identification: (**A**) identification of BrsC insect resistance; (**B**) resistance identification of BrsC35 inoculated with different test insects.

Three fresh, tender leaves were collected from each of 36 transgenic positive seedlings and placed in Petri dishes lined with moist filter paper, with one leaf per dish. Three second-instar *Helicoverpa armigera* larvae were inoculated onto each leaf. All the Petri dishes were incubated in an artificial climate chamber at 26–28 °C and a relative humidity of 60–80%. After 5 days of incubation, the leaves of the control cotton variety CCRI 24 were heavily consumed, and the larvae grew significantly. The leaves of the transgenic cotton lines BrsC02, BrsC35, BrsC17, BrsC31, BrsC36 and BrsC39 suffered feeding damage to varying degrees. Nevertheless, larval growth was inhibited after feeding, and all the larvae eventually died. Among these lines, BrsC35 showed the slightest leaf damage with only trace feeding; the larvae fed on BrsC35 hardly grew and died eventually (Figure 2A). The results indicated that the transgenic cotton line BrsC35 had strong resistance to the lepidopteran pest *Helicoverpa armigera*.

To further verify the insect resistance of the transgenic cotton line BrsC35, laboratory bioassays were conducted to evaluate its resistance to three lepidopteran pests: *Helicoverpa armigera*, *Ostrinia nubilalis* and *Spodoptera frugiperda*.

The results showed that the leaves of the control cultivar CCRI 24 were severely consumed and presented a sieve-like phenotype after inoculation with *H. armigera*, *O. nubilalis* and *S. frugiperda*. For the insect-resistant transgenic cotton line CCRI 127, only trace leaf feeding was observed, and all the inoculated larvae died. The transgenic cotton line BrsC35 exhibited a performance consistent with CCRI 127, with merely trace feeding damage and complete larval mortality (Figure 2, Table 1). It is indicated that BrsC35 possesses insect resistance comparable to CCRI 127, and was therefore selected as the research material for subsequent molecular characteristic analysis via genome sequencing.

### 2.3. Detection of Exogenous Protein Accumulation

The expression levels of the target protein in different tissues of transgenic cotton were determined by ELISA at the seedling stage, squaring stage, and flowering and boll stage. The results showed that the target protein exhibited the highest expression in leaves at the squaring stage, with an average expression level of approximately 1409.69 ng/g fwt. These results indicated that the target protein could be stably expressed at the translational level in the transgenic line BrsC35 (Table 2).

### 2.4. Molecular Characterization of Transgenic Cotton Line BrsC35 by PacBio Sequencing

The purity and integrity of the extracted genomic DNA from the transgenic cotton line BrsC35 were examined by agarose gel electrophoresis. The results showed that the concentration and integrity of the extracted DNA met the requirements for library construction. The total throughput of the third-generation sequencing data was 26.70 Gb (26,699,992,903 bp), with a total of 1,852,872 reads obtained. The maximum read length was 48,683 bp, and the minimum was 14,404 bp. These results indicate that the sequencing data were sufficient in quantity and of qualified quality.

**Figure 3:**
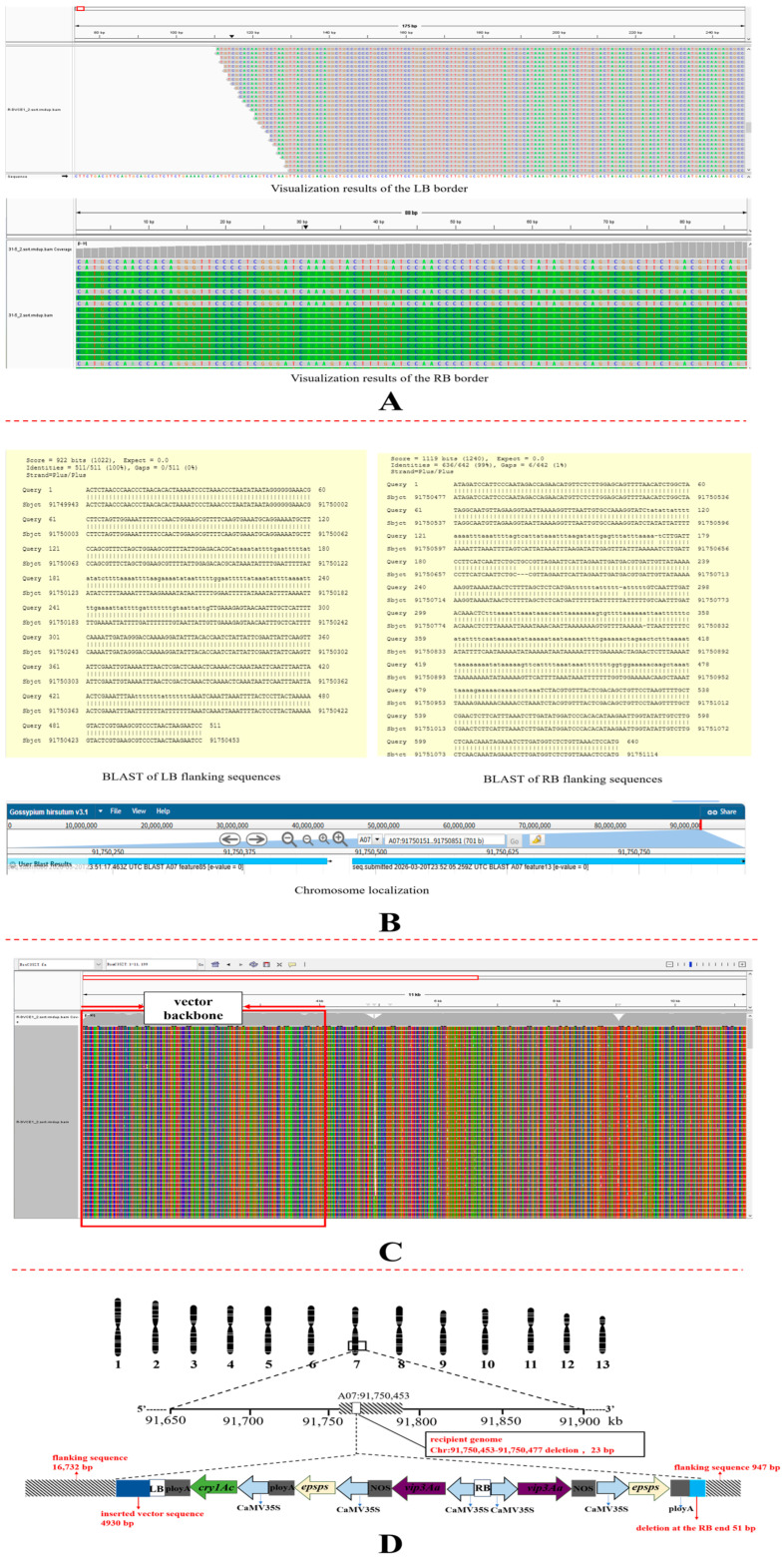
(**A**) Visualization analysis of LB and RB in transgene cotton BrsC35. (**B**) Chromosomal localization and flanking sequence alignment analysis of chromosome A07. (**C**) Visualization of the alignment of sequencing data with vector sequences using minimap2. (**D**) Analysis of the A07 integration site and the flanking sequence of the insert DNA fragment in the receptor genome of BrsC35.

Integration site analysis: Visualization analysis was performed by aligning the sequencing data with the exogenous insertion sequence using the minimap2 software. A total of 29 reads were mapped to the LB region of the T-DNA, whereas no junction region corresponding to the RB end was identified when aligned with the T-DNA region (Figure 3A). Flanking sequence analysis of the aligned reads on the PacBio alignment map of BrsC35 revealed that all these sequences could be mapped to the RB end of the T-DNA.

One of the sequences was selected for alignment against the cotton reference genome Gossypium hirsutum v3.1 in the Phytozome14 database. The results showed that the LB end of this sequence was adjacent to position 91,750,453 on chromosome A07 of the cotton genome (Figure 3B). Based on the cotton genome alignment, a 1508 bp left border flanking sequence and a 2230 bp right border flanking sequence were successfully obtained. The RB end of the inserted fragment was located adjacent to position 91,750,477 on chromosome A07. Since the 5′ end of the T-DNA was inserted upstream in the cotton genome, the T-DNA was determined to be integrated in the forward orientation. Further alignment with the T-DNA region revealed that the target genes *vip3A* and *epsps* were inserted into the cotton genome in the reverse orientation along with the T-DNA. At this insertion locus, the exogenous sequence contained one *cry1Ac*, two copies of *vip3A* and *epsps*, together with their corresponding promoters and terminators.

In this study, sequencing data were grouped and aligned individually to the vector sequence using the minimap2 software. The visualization analysis revealed that during genetic transformation, integration of the exogenous sequence into cotton chromosome A07 was accompanied by the insertion of a 4930 bp vector backbone segment into the recipient genome along with the T-DNA (Figure 3C).

In summary, based on precise alignment and analysis of the sequencing data, the exogenous insert in the transgenic cotton line BrsC35 was integrated into the cotton genome in multiple copies and multiple loci. Alignment showed that the exogenous sequence was inserted at positions 91,750,453–91,750,477 on cotton chromosome A07. The insertion caused a 23 bp deletion in the cotton genome (sequence: AACAATCATCCCAAAACTGGGCC). At the LB end, a 4930 bp vector backbone fragment was inserted together with the transgene, while a 51 bp deletion occurred at the RB end of the T-DNA. Based on the integration locus, T-DNA integrity, and flanking sequence information of the transgenic cotton line BrsC35, we constructed a schematic diagram of the exogenous gene integration at the A07 locus in the cotton genome (Figure 3D).

### 2.5. PCR Verification of BrsC35 Flanking Sequences

**Figure 4:**
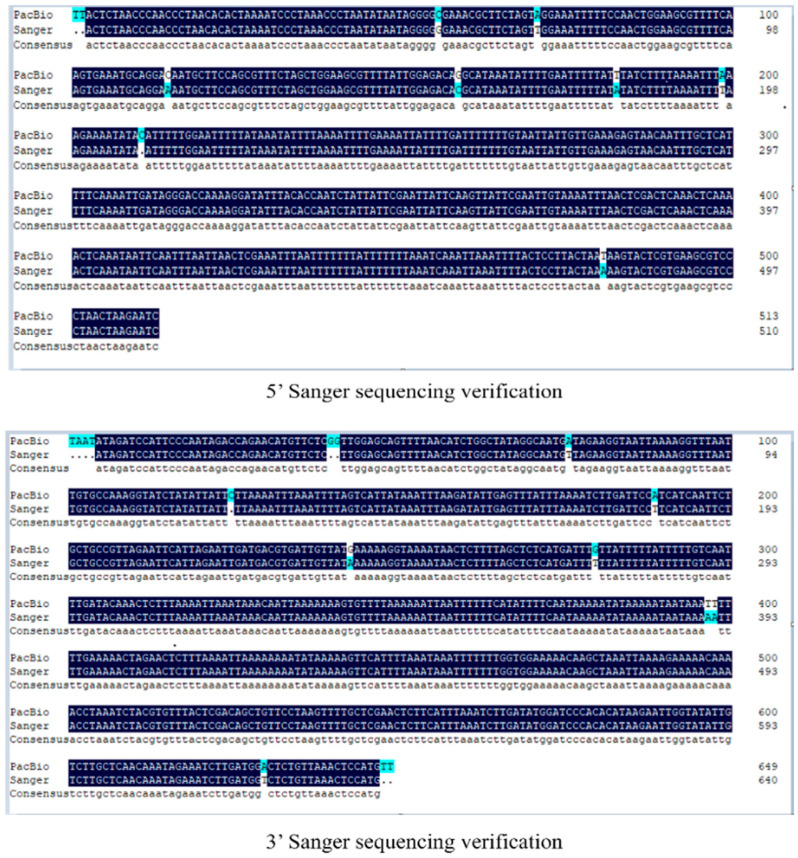
Verification of flanking sequences at the A07 locus.

In this study, PCR amplification was performed on the flanking sequences of the A07 loci in the transgenic cotton line BrsC35 obtained by PacBio sequencing, followed by Sanger sequencing. Analysis indicated that the flanking sequences of the transgenic cotton line BrsC35 obtained by PacBio sequencing were basically consistent with the Sanger sequencing results (Figure 4).

### 2.6. Establishment of a Specific PCR System for Transgenic Cotton Line BrsC35

(1) Establishment of A Transformant-Specific PCR Detection System for the LB End

**Figure 5:**
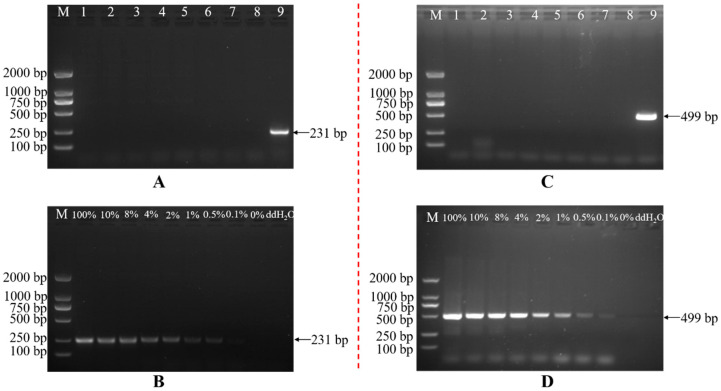
Establishment of a transformant-specific PCR detection system: (**A**) interspecific PCR amplification of left boundary specificity of transformant BrsC35; (**B**) the sensitivity detection for the left boundary of transformant BrsC35; (**C**) interspecific PCR amplification of right boundary specificity of transformant BrsC35; (**D**) the sensitivity detection for the right boundary of transformant BrsC35. M: Trans2K DNA Marker; 1: sorghum; 2: Arabidopsis thaliana; 3: rice; 4: tobacco; 5: soybean; 6: maize; 7: cotton 24; 8: ddH_2_O; 9: BrsC35.

In this study, a series of transformant-specific primers was designed based on the flanking sequence of the cotton genome at the left border of the insertion and the LB sequence of the insert obtained from high-throughput sequencing. PCR amplification was performed using genomic DNA from sorghum, *Arabidopsis thaliana*, rice, tobacco, soybean, maize, CCRI 24, and BrsC35 as templates. The results showed that the primer pair A07QTY-F1/A07QTY-R1 exhibited excellent specificity: only the BrsC35 sample yielded a specific target band of 231 bp (Figure 5A), whereas no target band was amplified in any other sample. The detection sensitivity reached 0.1% (Figure 5B).

(2) Establishment of a Transformant-Specific PCR Detection System for the RB End

A series of primers was designed based on the flanking sequence on the cotton genome at the right border of the insertion and the RB sequence of the insert obtained previously. PCR amplification was carried out using genomic DNA from sorghum, *Arabidopsis thaliana*, rice, tobacco, soybean, maize, CCRI 24, and BrsC35 as templates. The results showed that the primer pair A07-HTY-F1/A07-HTY-R2 exhibited excellent specificity: only the BrsC35 sample produced a specific target band of 499 bp (Figure 5C), while no target band was amplified in the other samples. The detection sensitivity reached 0.1% (Figure 5D).

### 2.7. Analysis of Genetic Stability of Target Genes in Transgenic Cotton Line BrsC35

**Figure 6:**
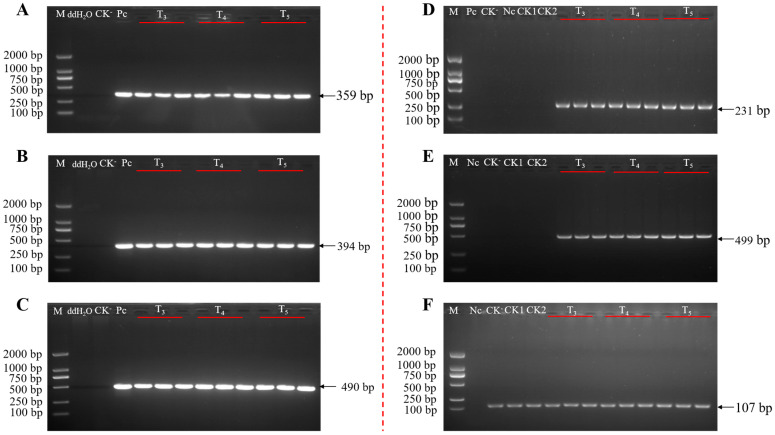
Target gene and event-specific PCR amplification in T_3_–T_5_ generations of the transgenic cotton line BrsC35: (**A**) the result of PCR for gene *epsps*; (**B**) the result of PCR for gene *cry1Ac*; (**C**) the result of PCR for gene *vip3A*. (**D**) Left boundary specificity detection; (**E**) right boundary specificity detection; (**F**) reference gene *sadI* detection. M: Trans2K DNA Marke; P: plasmid positive control; CK^-^: CCRI 24; Nc: control without DNA water; CK1: sister transformant BrsC02; CK2: sister transformant BrsC31; T_3_~T_5_: three generations of BrsC35, 3 plants per generation.

To determine whether the target genes *epsps*, *cry1Ac*, and *vip3A* were integrated into the genome of the recipient cotton, genomic DNA was extracted from fresh young leaves of T_3_- to T_5_-generation transgenic insect-resistant cotton plants. PCR detection was performed using primers specific for *epsps*, *cry1Ac*, and *vip3A*. Target bands were successfully amplified in the genomes of three consecutive generations of the BrsC35 transformant, demonstrating that the inserted sequence was stably integrated into the cotton genome and could be stably inherited across successive generations (Figure 6A–C).

To verify the stability of the insertion locus of the exogenous sequence in the recipient genome, three PCR-positive plants from each of the T_3_, T_4_, and T_5_ generations of BrsC35 were selected. Genomic DNA was extracted from leaves and used as templates for transformant-specific PCR detection. The results showed that all the T_3_ to T_5_ BrsC35 plants amplified a 231 bp left border fragment and a 499 bp right border fragment. No target bands were observed in negative controls, including water, the pC2300-VEC vector plasmid, other transformants derived from the same vector, and the recipient control CCRI 24. Meanwhile, the cotton endogenous reference gene *SadI* was successfully amplified, confirming the validity of the PCR system (Figure 6D,E). These results indicate that the insertion position of the exogenous sequence is stably inherited across generations of transformant BrsC35.

### 2.8. Analysis of Genetic Stability of Target Traits in Transgenic Cotton Line BrsC35

In this study, the gene function of the transgenic cotton line BrsC35 was evaluated starting from the T_1_ generation. The insect resistance of T_3_–T_5_-generation plants of the transgenic cotton line BrsC35 was determined via laboratory bioassay.

At the five-leaf stage of the transgenic cotton line BrsC35, three fresh tender leaves of BrsC35 and its receptor variety CCRI 24 were respectively placed in Petri dishes lined with moist filter paper, with one leaf per dish. Each dish was inoculated with three second-instar larvae of *Helicoverpa armigera*, three of *Ostrinia nubilalis*, and three of *Spodoptera frugiperda*. Larvae with good vitality were selected for inoculation. After inoculation, the Petri dishes were sealed and cultured in an artificial climate chamber at 26–28 °C with relative humidity of 60–80%.

**Figure 7:**
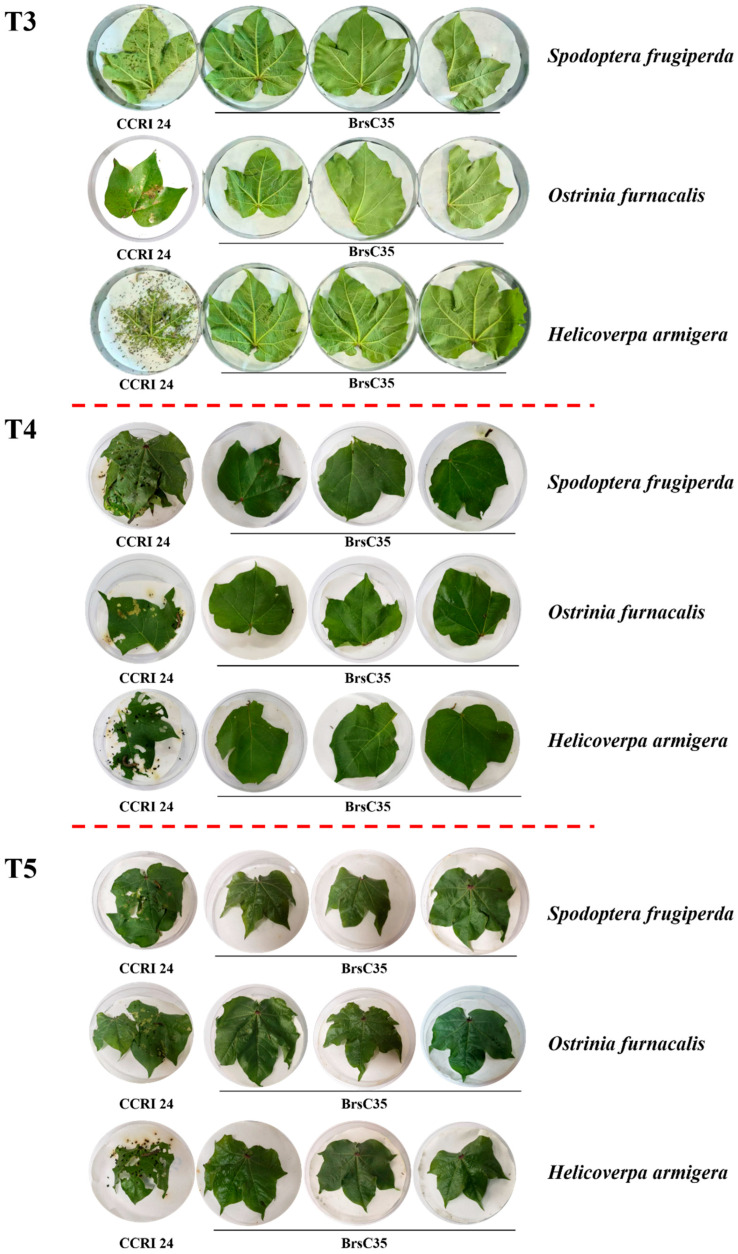
Evaluation BrsC35 of insect resistance at T_3_~T_5_ in the laboratory.

The phenotypic observation results showed that the leaves of the non-transgenic receptor CCRI 24 were heavily consumed by *Helicoverpa armigera*, *Ostrinia nubilalis* and *Spodoptera frugiperda*, while the leaves of BrsC35 remained intact and the inoculated larvae died (Figure 2, Figure 3, Figure 4, Figure 5 and Figure 6). It indicated that the transgenic cotton line BrsC35 exhibits insect resistance against lepidopteran pests including *Helicoverpa armigera*, *Ostrinia nubilalis* and *Spodoptera frugiperda* (Figure 7).

## 3. Discussion

With the continuous popularization and application of *Bt* transgenic cotton, the resistance issue of target pests of transgenic maize has attracted widespread attention. To ensure the sustainable promotion and application of transgenic maize and delay the development of resistance to *Bt* transgenic cotton in target pests, the implementation of resistance management strategies is particularly crucial. Resistance management strategies mainly adopt specific measures, such as the widely applied refuge strategy and pyramided gene strategy, to prevent or delay the occurrence of resistance in target pests. Nevertheless, given the long-term commercialization of transgenic insect-resistant cotton, *cry1Ac* has been the primary functional gene deployed against cotton bollworm. *Helicoverpa armigera* populations in major cotton-producing regions have evolved resistance genes, with resistant individuals even emerging in the field. To inhibit the reproduction and further evolution of resistant cotton bollworm populations on transgenic cotton, the combined application of pyramided multiple genes and refuge strategy can be adopted in future commercialization for more efficient control of cotton bollworm.

In terms of the analysis of insertion sites and flanking sequences, copy number identification, and vector backbone residue detection of genetically modified products, genome sequencing exhibits high accuracy and can detect insertion sites overlooked by conventional techniques such as TAIL-PCR and chromosome walking [46,47], as well as acquire complete molecular characterization information of complex transformants. For instance, in the transgenic maize line IE09S034, traditional methods including inverse PCR and TAIL-PCR failed to determine its insertion sites and flanking sequences. In contrast, genome sequencing not only successfully resolved its molecular characteristics but also identified the insertion of multiple unintended fragments [48]. More importantly, a single set of sequencing data can simultaneously provide all the genome-wide molecular characteristic information required for safety assessment, enabling a more comprehensive and higher-resolution analysis of genetic modifications integrated into the host genome [49].

## 4. Materials and Methods

### 4.1. Plant Materials, Plasmids and Strains

The recipient cotton variety used in this study was CCRI 24. The herbicide-tolerant gene *epsps* and insect-resistant genes *vip3A* and *cry1Ac* used in this study were codon-optimized by previous researchers in our laboratory and subsequently synthesized by Sangon Biotech (Shanghai) Co., Ltd., Shanghai, China. It should be emphasized that the *epsps* gene in this study was used only as a herbicide screening marker, rather than a functional gene. *Escherichia coli* competent cells DH5α were purchased from Nanjing Vazyme Biotech Co., Ltd., Nanjing, China. and Agrobacterium tumefaciens competent cells LBA4404 were purchased from Jiangsu Cowin Biotech Co., Ltd., Taizhou, China. The plant expression vector pC2300-VEC was preserved in our laboratory (WXJ Laboratory, Institute of Biotechnology, Chinese Academy of Agricultural Sciences). The vector backbone adopted in this study was pCambia2300, with the catalog number HG-VZC0330, which was provided by Solarbio Science & Technology Co., Ltd., Beijing, China. This vector carries kanamycin resistance and has a full length of 8742 bp.

### 4.2. Enzymes and Reagents

The enzymes and reagents used in this study are listed in Table 3.

### 4.3. Test Insects

The test insects used in this study are listed in Table 4.

### 4.4. Main Instruments

The main instruments used in this study included a PCR instrument, centrifuge, gel imaging system, etc. Detailed information is shown in Table 5.

### 4.5. Vector Construction and Genetic Transformation

The plant expression vector pC2300-VEC was constructed in our laboratory. The vector contains the codon-optimized herbicide-tolerant gene *epsps*, driven by the constitutive promoter CaMV 35S and terminated by a polyA signal; the codon-optimized insect-resistant genes *cry1Ac* and *vip3A* are both driven by the constitutive promoter CaMV 35S and terminated by the nos terminator. The plant expression vector pC2300-VEC was transformed into *Agrobacterium tumefaciens* strain LBA4404 by the freeze–thaw method. A single colony of *A. tumefaciens* LBA4404 harboring pC2300-VEC was picked and cultured overnight at 28 °C in YEB medium. Then, 1 mL of the bacterial suspension was inoculated into 100 mL fresh YEB medium and incubated at 28 °C for 4–6 h until the OD_600_ reached 0.6–0.8 for subsequent use. Using immature embryos as explants, the T-DNA region of pC2300-VEC was introduced into the genome of the cotton cultivar CCRI 24 via the *Agrobacterium*-mediated transformation method, and regenerated plants were obtained.

Cotton genetic transformation was entrusted to Kejidalong (Beijing) Biotechnology Co., Ltd., Beijing, China for completion.

### 4.6. Preparation of Culture Medium

YEB: Add 800 mL of deionized water into a beaker, then sequentially add 5 g of peptone, 5 g of sucrose, 1 g of yeast extract and 0.98 g of MgSO_4_. Adjust the pH to 7.0, and supplement with deionized water to a final volume of 1 L. Divide the solution equally into five 250 mL conical flasks, perform high-pressure sterilization at 120 °C, and store the medium in a refrigerator at 4 °C.

### 4.7. Screening of Transgenic Positive Plants and Identification of Insect Resistance

Detection primers were designed based on the exogenous target fragments. Using the extracted cotton genomic DNA as the template, genes *cry1Ac*, *epsps*, and *vip3A* were detected with the primer pairs JC-Cry1Ac-F1/JC-Cry1Ac-R1, JC-Epsps-F1/JC-Epsps-R1, and JC-Vip3A-F1/JC-Vip3A-R1 (Table 6), respectively. In this study, indoor bioassays were performed at the squaring stage on T_1_ transgenic insect-resistant cotton plants identified by PCR. The PCR reaction system was set to a total volume of 30 µL, containing 1.0 µL of genomic DNA, 1.0 µL of forward primer, 1.0 µL of reverse primer, 15 µL of 2× PCR mix, and 12 µL of ddH_2_O. The PCR thermal procedure was as follows: initial denaturation at 95 °C for 5 min; followed by 30 cycles of denaturation at 95 °C for 30 s, annealing at 62 °C (primer-dependent) for 30 s, and extension at 72 °C for 1 min; a final extension was performed at 72 °C for 5 min. The 2nd to 4th fully expanded leaves from the apex were selected. One piece of filter paper was placed at the bottom of a Petri dish with 600 µL of sterile water to keep the cotton leaves from drying during the test. Leaves were placed in Petri dishes, and five 1-day-old larvae were inoculated on each leaf sample. Petri dishes were sealed with tape after inoculation and incubated in an artificial climate chamber at 26–28 °C and 60–80% relative humidity (RH). On the 5th day after inoculation, leaf feeding damage was observed, larval survival was recorded, and larval mortality and corrected mortality were calculated.

### 4.8. Expression Analysis of Target Protein in Transgenic Cotton

To detect the expression level of the target gene in the BrsC35 transgenic line, fresh leaves were collected at the seedling, squaring and boll stages. The expression content of the target protein was determined by ELISA, with three biological replicates and three technical replicates set in this study. Refer to the instructions of VIP3A ELISA Quantitative Detection Kit (Cat. No. AA1641) and Cry1Ac ELISA Quantitative Detection Kit (Cat. No. AA0341) for the detailed procedures.

### 4.9. Molecular Characterization of Transgenic Cotton Line BrsC35 via PacBio Sequencing

Molecular characteristics of the transgenic cotton line BrsC35 were analyzed using third-generation sequencing technology on the PacBio Sequel II/IIe platform with a sequencing depth of 70×. The detailed procedures are as follows:

(1) DNA Extraction

Leaves were collected from the field-grown transgenic insect-resistant cotton *BrsC35*. Genomic DNA was extracted using a commercial kit: Rapid Plant Genomic DNA Extraction Kit (Non-spin Column), Cat. No. DP321 (Tiangen Biotech Co., Ltd., Beijing, China). The purity of genomic DNA extracted from the transgenic cotton line BrsC35 was determined using a spectrophotometer. Meanwhile, the DNA sample was diluted to an appropriate concentration and detected via agarose gel electrophoresis. Clear bands without smearing and degradation phenomena indicate excellent DNA integrity.

(2) Sequencing Library Construction

One library was constructed for the transgenic cotton line BrsC35. Qualified DNA was sheared into 15–18 kb fragments using a Covaris focused-ultrasonicator. Large DNA fragments were enriched and purified with magnetic beads. The fragmented DNA was subjected to DNA damage repair and end repair. SMRTbell dumbbell-shaped adapters were ligated to both ends of the DNA fragments, and unligated fragments were removed by exonuclease digestion. Detailed procedures followed the instructions of the SMRTbell Express Template Prep Kit 2.0 (Pacific Biosciences of California, Inc., Menlo Park, CA, USA).

(3) Sequencing Strategy

The constructed library was sequenced on a PacBio Sequel II/IIe system in CCS (Circular Consensus Sequencing) mode using one SMRT Cell. Sequencing was performed by Novogene Co., Ltd. (Beijing, China).

(4) Data Quality Control

Raw Polymerase reads (adapter-flanked dumbbell structures) were processed to remove adapters and generate subreads. Subreads were filtered with a minimum length threshold of 50 bp. High-accuracy HiFi reads were generated using the ccs program with parameters:

min-passes = 3, min-rq = 0.99, ensuring all the reads had a quality score above Q20.

(5) Data Analysis

Format conversion: HiFi reads in BAM format were converted to FASTQ format using bam2fasta from the SMRT Link software (V25.1) package. Sequence alignment: Sequencing reads were aligned to the vector sequence using minimap2 (v2.26-r1175). Indexing: Visualizable indices were built with samtools index to generate file.rmdup.sort.bam.bai. Visualization: Comparative analysis was performed using IGV 2.5.0 (Integrative Genomics Viewer). The insertion profile of exogenous genes in the cotton genome was analyzed to determine the copy number of transgenes and the presence or absence of vector backbone contamination.

### 4.10. PCR Identification of Flanking Sequences in Transgenic Cotton Line BrsC35

In this study, the primers A07-QF1/A07-QR2 and A07-HF1/A07-HR1 were designed based on the two flanking sequences of the transgenic cotton line BrsC35 obtained from genome sequencing and bioinformatics analysis, as well as the sequence of the transformation vector pC2300-VEC. The primer sequences are shown in Table 6. Genomic DNA of cotton was extracted using a novel plant genomic DNA extraction kit for PCR amplification. The PCR reaction system and procedure are detailed in Section 2.7. The DNA fragments amplified by PCR were recovered after 0.8% agarose gel electrophoresis and subjected to Sanger sequencing. The sequencing results were aligned and verified with the flanking sequences obtained from whole-genome sequencing.

### 4.11. Establishment of a Specific PCR Detection System for Transgenic Cotton Line BrsC35

(1) Establishment of a Left Border-Specific PCR Detection System for Transformant BrsC35

In this study, multiple primer pairs were designed separately in the 5′ flanking sequence and the T-DNA region of the transgenic cotton. After primer combination screening, the primer pair 5′-QF1 (5′-CGAATTGTAAAATTTAACTCGACTCA-3′) and 5′-QR1 (5′-AGCCTGAATGGCGAATGCTAGAGCAG-3′) was finally selected as the event-specific detection primer for the 5′ flanking sequence of transgenic insect-resistant cotton BrsC35, with an amplified fragment size of 231 bp.

Interspecific specificity analysis:

PCR amplification was performed using the screened primer pair 5′-QF1/5′-QR1 with genomic DNA from *sorghum*, *Arabidopsis thaliana*, *rice*, *tobacco*, *soybean*, *maize*, *CCRI 24*, and BrsC35 as templates. The specificity of the primer pair 5′-QF1/5′-QR1 was examined by 1.2% agarose gel electrophoresis.

Sensitivity test of the specific detection primer:

Genomic DNA from the recipient CCRI 24 and transgenic cotton line BrsC35 was mixed to form a serial gradient of mass percentages: 100%, 10%, 8%, 4%, 2%, 1%, 0.5%, 0.1%, and 0%. Using these mixtures as templates, PCR amplification was conducted with the primer pair 5′-QF1/5′-QR1.

(2) Establishment of a Right Border-Specific PCR Detection System for Transformant BrsC35

In this study, multiple primers were designed in the 3′ flanking sequence and the T-DNA region of the transgenic cotton. After screening of primer combinations, the primer pair 3′-HF1 (5′-ATACAAAGGCAGCATAATAGCTCGAG-3′) and 3′-HR1 (5′-TCTAACGGCAGCAGAATTGATGAAGG-3′) was finally selected as the event-specific detection primer pair for the 3′ flanking sequence of transgenic insect-resistant cotton BrsC35, with an amplified fragment size of 499 bp.

Interspecific specificity analysis:

PCR amplification was performed using the screened primer pair 3′-HF1/3′-HR1 with genomic DNA from sorghum, Arabidopsis thaliana, rice, tobacco, soybean, maize, CCRI 24, and BrsC35 as templates. The specificity of the primer pair 3′-HF1/3′-HR1 was examined by 1.2% agarose gel electrophoresis.

Sensitivity test of the specific detection primer:

Genomic DNA from the recipient CCRI 24 and transgenic cotton line BrsC35 was mixed to prepare gradient DNA solutions with mass percentages of 100%, 10%, 8%, 4%, 2%, 1%, 0.5%, 0.1%, and 0%. Using these mixtures as templates, PCR amplification was conducted with the primer pair 3′-HF1/3′-HR1.

## 5. Conclusions

In this study, molecular characterization of the transgenic cotton line BrsC35 was obtained using PacBio sequencing combined with Sanger sequencing. The exogenous sequence was inserted at positions 91,750,453–91,750,477 on chromosome A07 of the cotton genome, and a 23 bp fragment (AACAATCATCCCAAAACTGGGCC) of the cotton genome was deleted during the insertion process. A 4930 bp vector sequence was inserted at the left border (LB) of the exogenous gene, whereas a 51 bp deletion occurred at the right border (RB).

In addition, an event-specific detection method for the transgenic cotton line BrsC35 was established based on the known flanking sequence information, with a detection sensitivity of 0.1%, equivalent to 20 copies. The genetic stability of the target gene in the transgenic cotton line BrsC35 was evaluated from the T_3_ to T_5_ generations using event-specific PCR. Furthermore, the stability of the target trait of BrsC35 was assessed by infesting T_3_- to T_5_-generation plants with *Helicoverpa armigera*, *Ostrinia furnacalis* and *Spodoptera frugiperda*.

In summary, an insect-resistant transgenic cotton line was successfully developed in this study, and the complete molecular characterization of the transgenic cotton line BrsC35 was accurately obtained using PacBio sequencing combined with Sanger sequencing.

## Figures and Tables

**Table 1:** Analysis of insect resistance of BrsC35 by laboratory bioassay.

Investigation Time	Treatment	*Helicoverpa armigera*		*Ostrinia furnacalis*		*Spodoptera frugiperda*	
		Mortality (%)	Calibrated Mortality (%)	Mortality (%)	Calibrated Mortality (%)	Mortality (%)	Calibrated Mortality (%)
1 d	BrsC35	11.11 ± 19.24	0.00 ± 0.00	0.00 ± 0.00	0.00 ± 0.00	22.22 ± 19.24	0.00
	CCRI 127	11.11 ± 19.24	0.00 ± 0.00	11.11 ± 19.24	0.00 ± 0.00	0.00 ± 0.00	0.00 ± 0.00
	CCRI 24	0.00 ± 0.00	-	0.00 ± 0.00	-	0.00	-
3 d	BrsC35	33.33 ± 0.00	33.33 ± 0.00	22.22 ± 19.24	22.22 ± 19.24	44.44 ± 38.49	21.05 ± 33.33
	CCRI 127	33.33 ± 0.00	33.33 ± 0.00	11.11 ± 19.24	0.00 ± 0.00	33.33 ± 0.00	33.33 ± 0.00
	CCRI 24	0.00 ± 0.00	-	0.00 ± 0.00	-	-	0.00 ± 0.00
5 d	BrsC35	77.78 ± 19.24	77.78 ± 19.24	66.67 ± 0.00	66.67 ± 0.00	77.78 ± 19.24	72.73 ± 15.79
	CCRI 127	66.67 ± 0.00	66.67 ± 0.00	66.67 ± 0.00	66.67 ± 0.00	66.67 ± 0.00	66.67 ± 0.00
	CCRI 24	0.00 ± 0.00	-	0.00 ± 0.00	-	0.00 ± 0.00	-
7 d	BrsC35	100.00 ± 0.00	98.15 ± 0.00	100.00 ± 0.00	100.00 ± 0.00	100.00 ± 0.00	100.00 ± 0.00
	CCRI 127	100.00 ± 0.00	100.00 ± 0.00	100.00 ± 0.00	100.00 ± 0.00	100.00 ± 0.00	100.00 ± 0.00
	CCRI 24	0.00 ± 0.00	-	11.11 ± 0.00	-	0.00 ± 0.00	-

**Table 2:** Target protein expression in different tissues and growth stages of BrsC35.

Stages	Tissues	Protein Classification	Protein Content (ng/g fwt)	
			BrsC35	CCRI 24
seedling stage	leaf	Vip3A	937.45 ± 56.74	NA
		Cry1Ac	1074.84 ± 17.07	NA
squaring stage	leaf	Vip3A	1409.69 ± 73.59	NA
		Cry1Ac	1155.96 ± 44.81	NA
	flower bud	Vip3A	774.28 ± 47.16	NA
		Cry1Ac	604.16 ± 19.82	NA
flowering and boll stage	leaf	Vip3A	1306.85 ± 12.04	NA
		Cry1Ac	1143.43 ± 40.79	NA
	cotton boll	Vip3A	607.32 ± 14.67	NA
		Cry1Ac	421.21 ± 10.54	NA

Note: NA = not analyzed; fwt = tissue fresh weight. Values in the table are presented as mean ± standard deviation.

**Table 3:** Enzymes and reagents.

Enzymes/Reagents	Source
Taq polymerase, restriction enzymes	New England Biolabs, Ipswich, MA, USA
Trans DNA Marker	TransGen Biotech, Beijing, China
Phanta Max DNA Polymerase	Vazyme Biotech, Nanjing, China
Amp	BioDee, Beijing, China
DH5α competent cells	CoWin Biotech, Taizhou, China
Plant Genome DNA Extraction Kit	Tiangen Biotech, Beijing, China
ELISA Kit	YouLong Biotech, Shanghai, China
Agarose Gel Extraction Kit	Majorbio, Shanghai, China
Conventional chemicals	BUCT, Beijing, China

**Table 4:** Experimental insects.

Test insects	Source
*Spodoptera frugiperda*	Meiyan Agricultural Tech., Beijing, China
*Helicoverpa armigera*	Meiyan Agricultural Tech., Beijing, China
*Ostrinia furnacalis*	Meiyan Agricultural Tech., Beijing, China

**Table 5:** Main instruments.

Name	Source
Room-temperature centrifuge	Thermo D-37520, Waltham, MA, USA
Vortex mixer	DAIHAN Scientific, Seoul, Republic of Korea
Ice maker	Snowke Electric Co., Ltd., Hefei, China
Constant temperature shaker	THZ-C, Peiying, China
Constant temperature incubator	DH3600A, Taisite Instrument Co., Ltd., Tianjin, China
Gel imaging system	G:BOX, Syngene, Cambridge, UK
PCR amplifier	BIO-RAD ALS1296, Hercules, CA, USA
Artificial climate chamber	Dongnan Instrument Co., Ltd., Ningbo, China
Covaris M220 Focused-ultrasonicator	Covaris, Woburn, MA, USA
NanoDrop 2000 Micro-volume UV-Vis Spectrophotometer	Thermo Scientific, Waltham, MA, USA

**Table 6:** Primers’ name and sequence information.

Primers Name	Primer Sequences (5′ to 3′)	Target Amplified Band Size/bp
JC-Cry1Ac-F1	TGGATAGGAACCTCGATGTAACCACG	394 bp
JC-Cry1Ac-R1	TCGCCTACGGTACTTCTTCCAACCTG	
JC-Epsps-F1	CGTCCTCACCCTCCAGAAGTCC	359 bp
JC-Epsps-R1	CGTCGCTTTCCCAGTCACCAAG	
JC-Vip3A-F1	AGATGATGAAGTTATCGCCCCAGGCT	490 bp
JC-Vip3A-R1	CTCATCACCCTCACTTGCAAGTCAT	
A07QTY-F1	CGAATTGTAAAATTTAACTCGACTCA	231 bp
A07QTY-R4	AGCCTGAATGGCGAATGCTAGAGCAG	
A07-HTY-F1	ATACAAAGGCAGCATAATAGCTCGAG	499 bp
A07-HTY-R2	TCTAACGGCAGCAGAATTGATGAAGG	
A07-QF1	ACTCTAACCCAACCCTAACACACTA	800 bp
A07-QR2	ATGATTACGAATTCGAGCTCGGTAC	
A07-HF1	ATACAAAGGCAGCATAATAGCTCGAG	934 bp
A07-HR1	TCGCCAGCTGGCGTAATAGCGAAGA	
Sad1-F	CCAAAGGAGGTGCCTGTTCA	107 bp
Sad1-R	TTGAGGTGAGTCAGAATGTTGTTC	

## Data Availability

All data generated or analyzed during this study are available from the corresponding author upon reasonable request.
